# Development of a highly sensitive colloidal gold semiquantitative method for the determination of difenoconazole residues in citrus

**DOI:** 10.3389/fnut.2024.1341219

**Published:** 2024-03-25

**Authors:** Ruobing Wang, Min Gong, Yang Liu, Weiran Zhu, Kai Zhang, Yidi Zhao, Chen Yin, Yuan Liu, Jian Wang, Yuping Wan

**Affiliations:** ^1^Hebei Key Laboratory of Quality & Safety Analysis-Testing for Agro-Products and Food, Hebei North University, Zhangjiakou, Hebei, China; ^2^Hainan Inspection and Detection Center of Modern Agriculture, Haikou, Hainan, China; ^3^Beijing Kwinbon Technology Co., Ltd., Beijing, China; ^4^Beijing Engineering Research Centre of Food Safety Immunodetection, Beijing, China

**Keywords:** difenoconazole, detection limit, gold nanoparticles, colloidal gold immunochromatographic technique, rapid diagnosis strips

## Abstract

**Introduction:**

Difenoconazole (DIFE) is a common pesticide used in citrus cultivation; excessive intake can cause neurological damage to the organism, and the existing colloidal gold immunochromatographic test strips cannot meet the requirements for the detection of citrus samples.

**Methods:**

Difenoconazole test strip was prepared based on the colloidal gold immunochromatographic technique (GICT), and its application in citrus samples was investigated; with colloidal gold (CG) as the probe, the optimization of GICT parameters, and the determination of reaction method, the immunochromatographic test strips for the detection of DIFE in citrus was developed, and the limit of detection (LOD), specificity, accuracy, and stability of the test strips were verified.

**Results:**

The results showed that the visual detection limit of the prepared colloidal gold immunochromatographic test strips was 0.2 mg/kg and the quantitative range was 0.06–0.6 mg/kg, and the test strips could specifically identify DIFE and have no cross-reaction with other common triazole pesticides. The detection method established in this study was verified by the GC–MS method, and the detection results achieved good consistency (*R*^2^ > 0.98).

**Conclusion:**

The test strips developed in this study have good performance and can be used for highly sensitive detection of citrus samples.

## 1 Introduction

Difenoconazole (DIFE) is a broad-spectrum, high-efficiency triazole systemic fungicide, which inhibits the growth of fungi by inhibiting the germination of pathogen spores, leading to their death, and has a preventive effect on vegetables, fruit trees, cereals, and other crops, with high adhesion and low degradability (1). In view of the high risk of DIFE to fish (2) and mammals (3) and its high levels in indoor dust (4) and water bodies (5), to prevent excessive intake of DIFE by humans, the residue limit of DIFE has been clearly set in GB.2763–2021 National Food Safety Standard - Maximum Residue Limit of Pesticides in Foods (6) issued by China, which is 0.01 mg/kg per person as the tolerable daily intake. Citrus is the main fruit crop, with an annual production of more than 100 million tons, and the production of oranges and tangerines alone reaches more than 50 million tons (7), making it one of the most vital cash crops in the world (8). Citrus is the most exported fruit in China (9), but with the continuous increase in citrus commercial planting area and yield, citrus diseases and pests, especially diseases, have increased gradually, and the problem of citrus simultaneously suffering from multiple diseases appears which seriously affects industrial development. DIFE is a specific agent for the prevention and treatment of some common diseases, such as citrus anthrax, citrus scab, and citrus sarcoidosis, but there are great food safety hazards associated with the high rate of DIFE exceedance due to excessive spraying, so testing DIFE residues in citrus is necessary.

At present, some studies have reported that classical chemical technologies employed for monitoring the difenoconazole residue, such as gas chromatography-tandem mass spectrometry (GC–MS) (10), the limit of detection (LOD) for difenoconazole was 0.02 mg/kg. The trace detection method for the determination of residues of difenoconazole in a plant sample by high-performance liquid chromatography–tandem mass spectrometry (HPLC-MS/MS) was developed (11). The detection limits for difenoconazole were 0.0002–0.0004 mg/kg, and the quantitation limits were 0.0044–0.011 mg/kg in citrus leaves and whole fruits. An effective method was developed to determine difenoconazole residues in pollen and honey of litchi by modified QuEChERS-HPLC-MS/MS (12). The LODs of difenoconazole were 0.25 μg/kg and 0.50 μg/kg. Although the chemical analytical methods are accurate, they are limited to laboratory analysis. Correct experimental results still need complex sample preparation methods, long detection time, and well-skilled personnel (13).

In addition to instrumental analysis methods, immunological methods have also been used to determine difenoconazole residues since such techniques are simple and sensitive (14). Immunoassays have been widely used to detect various analytes in biomedicine, food, and the environment because of the advantages of being simple, fast, and economical (15). Chen (16) established an indirect competitive enzyme-linked immunosorbent assay (ic-ELISA). Under this condition, the linear detection range (IC_20_ ~ IC_80_) of difenoconazole was 0.49 ~ 3.90 ng / mL, and the LOD was 0.33 ng/mL. A surface-enhanced Raman spectroscopy (SERS) has been reported to detect difenoconazole in banana (17). The results indicated a detection limit of 0.16 mg/kg in banana. However, the long reaction time, tedious operating procedures, and the need for specialized equipment have limited the application of ELISAs in field testing (18).

Compared with other detection methods, the GICT has low requirements on the purity of the sample, does not require complex sample pre-treatment, and is simple to operate during the experiment. It is less dependent on the instrument and is suitable for public use. In addition, it has low cost, short detection time, intuitive detection results, and can be detected on-site (19). At present, GICT is widely used in many fields such as food (20–22), clinical medicine (23, 24), environmental analysis (25, 26), and biology (27). However, in the application of rapid detection in food, trace substances to be detected usually exist in complex food matrices, which pose great challenges to traditional CG-ICA in terms of sensitivity and matrix tolerance (28). The extraction of pesticide residues in samples with organic solvents affects the chromatographic effect of the NC membrane and causes environmental pollution (29). The report on the analysis of DIFE based on the colloidal gold immunochromatographic technique is that Tingting Cui (30) used the colloidal gold immunochromatographic method to detect DIFE in fruits and vegetables, resulting in a detection limit of the test strip at 0.5 mg/kg, which could not meet the detection of citrus samples. Cai (31) reduced the sensitivity of aflatoxin B_1_ from 0.5 μg/L to 0.05 μg/L based on the lyophilized gold reaction form, with a 10-fold increase in sensitivity. The lyophilized gold was the gold standard antibody dispensed in the microtiter plate. In the reflective form of lyophilized gold, the gold-labeled antibody was fully recognized with the antigen, and the amount of antigen binding to the T-line was reduced, resulting in a lighter T-line. Since the binding site of the gold-labeled antibody with the secondary antibody was different from the antigen–antibody binding site, the reflective form of lyophilized gold did not affect the color development effect of the C-line, thus achieving the purpose of reducing the detection limit (32).

In this study, a semiquantitative colloidal gold immuno-chromatographic test strip analysis method was established with citrus as the sample and compared with the detection method specified in the national standard (33), demonstrating that the developed colloidal gold test strips were accurate, rapid, convenient, conducive to ecological protection, and could be used for the quantitative detection of large batches of samples.

## 2 Materials and methods

### 2.1 Materials

DIFE, Triadimenol, Triadimefon, Tebuconazole, and Myclobutanil standards (≥99%) were purchased from Dr. Ehrenstorfer, Germany; potassium carbonate, sucrose, phosphate, sodium carbonate, trisodium citrate (C_6_H_5_Na_3_O_7_), chloroauric acid (HAuCl_4_), and bovine serum albumin (BSA) were purchased from Sinopharm Chemical Reagent Co., Ltd.; sheep anti-mouse IgG, difenoconazole antigen, and difenoconazole monoclonal antibody were prepared by Beijing Bontem Biotechnology Co., Ltd.; nitrocellulose membrane CN140 was purchased from Millipore, USA; sample pads, gold standard pads, blotting paper, and backing plate were purchased from Shanghai Gold Standard Biotechnology Co., Ltd.; Citrus samples were purchased from supermarkets in Changping District, Beijing, China.

ESJ110-4A electronic balance (0.01 mg) was purchased from Shenyang Longteng Electronics Co., Ltd.; electrothermal magnetic stirrer was purchased from WIGGENS, Germany; SP-756P UV–Vis spectrophotometer was purchased from Shanghai Spectrum; H2100R high-speed frozen centrifuge was purchased from Hunan Xiangli Scientific Instruments Co., Ltd.; HM3035 gold spray film scribing instrument, CTS300 CNC cutting machine, and ZQ2402 microcomputer automatic chopping machine were purchased from Shanghai Gold Standard Biotechnology Co., Ltd.; GT-710 nano gold immunoassay reader was purchased from Beijing Kwinbon Technology Co., Ltd.; high-performance liquid chromatograph (UltiMate3000) was purchased from Thermo Fisher, USA; JSM 6701F scanning electron microscope was purchased from Japan Electronics.

### 2.2 Methods

#### 2.2.1 Preparation of colloidal gold

The traditional trisodium citrate method (34) was used for the preparation: 1 mL of 1% concentration of the chloroauric acid solution was added to 98 mL of ultrapure water with stirring at a constant speed until boiling, followed by the rapid addition of 2 mL of 1.5% trisodium citrate solution; the rotational speed was accelerated and the solution was kept boiling for approximately 10 min, during which the solution gradually changed from colorless to yellow and finally to purple red. After 10 min, the heating was stopped, and the rotational speed was maintained until the solution temperature decreased to normal temperature, followed by filtrating with a 0.22-μm filter, and the solution was sealed with sealing film.

#### 2.2.2 Identification of colloidal gold

The colloidal gold used the observation method, transmission electron microscopy method, and ultraviolet–visible spectrophotometer for identification. First of all, the color of colloidal gold was clear, transparent, and no particulate matter appears was observed; it indicated that colloidal gold was successfully fired. After a period of time, whether there was wall hanging, precipitation, flocculent, and other phenomena in the bottle was observed; if not, it indicated that the preparation of colloidal gold was stable. Then, the wavelength scanning of the gold particles was carried out by the ultraviolet–visible spectrophotometer to observe the peak width and the highest absorption peak, according to which the size and shape of the gold particles were calculated. Finally, scanning electron microscope (SEM) observation was the most intuitive way to determine the size and state of colloidal gold particles.

#### 2.2.3 Preparation of gold standard antibodies

The key for gold nanoparticles to couple with antibodies and form a stable colloidal state lies in the pH of the colloidal gold solution, which is generally adjusted with HCl solution or K_2_CO_3_ solution. When the solution pH is adjusted to near the antibody isoelectric point, on the one hand, the gold nanoparticles are in equilibrium with the electrostatic and hydrophobic properties of the antibody proteins so that the nanoparticles remain colloidal and no aggregation or sedimentation phenomenon occurs; on the other hand, the antibody can be maximally adsorbed on the surface of gold nanoparticles, thus reducing the specific or non-specific binding of gold nanoparticles to other proteins and improving the gold standard antibody specificity (35).

##### 2.2.3.1 Optimal labeling pH

In a centrifuge tube, 1 mL of colloidal gold solution was added, and the pH of the solution was adjusted with 0.2 mol/L K_2_CO_3_. Subsequently, 10 μL of DIFE antibody was added into each tube, and the solution was mixed and stood for 1 h at room temperature, and then 200 μL of 10% NaCl was added and stood for 2 h. The color change of the colloidal gold solution was observed. K_2_CO_3_ with less dosage and no color change in the solution is the pH optimum.

##### 2.2.3.2 Optimal amount of labeled protein

In a centrifuge tube, 1 mL of colloidal gold solution was taken, and 1.5 μL of 0.2 mol/L K_2_CO_3_ solution was added; 0.5, 1, 1.5, 2, 2.5, and 3 μL of DIFE antibodies were added, respectively. The solution was left at room temperature for 1 h, while 200 μL of 10% NaCl was added and left overnight to observe the color change of the colloidal gold solution. The optimal amount of protein labeling was determined by the small amount of protein and no color change in the solution.

##### 2.2.3.3 Optimal blocking solution

In a centrifuge tube, 2 mL of colloidal gold solution was taken, 3 μL of 0.2 mol/L K_2_CO_3_ solution was added, and then the determined DIFE antibody was added and allowed to stand for 10 min, waiting for the gold particles to couple with the protein. To ensure the specificity of the test strips and prevent non-specific binding, the other binding sites of the gold particles should be blocked with a blocking solution. In the centrifuge tube, 10% BSA, BSAH, and PEG 20000 were added, shaken well, and left for 10 min. The supernatant was removed by centrifugation at a speed of 10,000 r/min for 5 min at 4°C, the precipitation was retained, and 200 μL suspension was added and shaken for further use. The optimal blocking solution was judged by the color development status of the T and C lines.

#### 2.2.4 Reaction method determination

**Figure 1:**
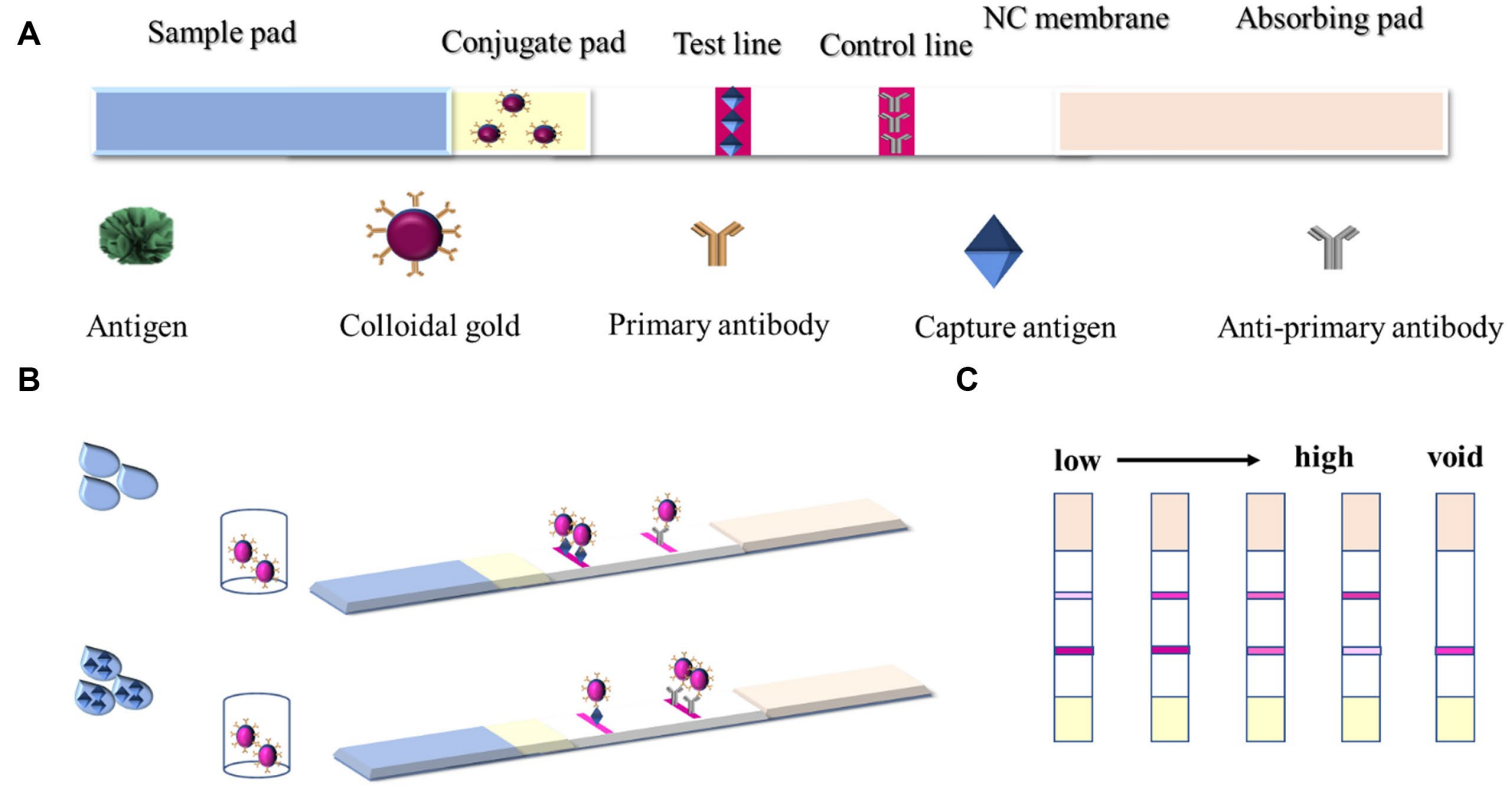
**(A)** Materials of the GICT system; **(B)** Colloidal gold immunochromatographic strip results; and **(C)** result determination.

The basic materials of the GICT system based on AuNFs as probes are shown in Figure 1A.

GICT consists of four top-down parts: absorbent paper, NC film, gold standard pad, and sample pad. The gold standard pad was the glass fiber pad carrying the gold standard antibody, and the sprayed gold was the gold standard antibody evenly sprayed on the gold standard pad. The DIFE standard or sample solution was dropped into the prepared sample pad, and the results were provided within 10 min through capillary action and immune reaction. The test strips were prepared using the principle of competitive inhibition. When there is no DIFE in the sample or its concentration is below the LOD, the AuNFs-labeled mAb would bind to the coating antigen on the T line and the goat anti-mouse IgG on the C line, which generates the darker red T line and the red C line. With a gradual increase in the amount of DIFE in the test sample, the binding between AuNFs-labeled mAb and coating antigen is gradually inhibited by DIFE, so the red T line would gradually become lighter (Figure 1B). Determined results are shown in Figure 1C.

To prepare test strips that could satisfy citrus detection, the two methods were compared. The optimal reaction method was judged by comparing the inhibition ratio of standards at different concentrations. The AuNFs were prepared according to Table 1.

**Table 1:** Choice of test strip reflection forms (*n* = 3).

Experimental methods	Concentration of the standard (mg/kg)	T/C values	Inhibition rate
Sprayed gold	0	2.256 ± 0.04	0
	0.25	0.194 ± 0.01	91.40%
	0.1	0.479 ± 0.05	78.77%
Lyophilized gold	0	4.313 ± 0.12	0
	0.25	0.264 ± 0.02	93.87%
	0.1	0.368 ± 0.04	91.47%

#### 2.2.5 Determination of colloidal gold immunochromatographic test strip results

To the prepared lyophilized microtiter reagent, 100 μL of sample extract to be tested or diluted difenoconazole extract was added; after 5 min incubation, all of them were pipetted into the prepared test strips, and then the test strips were observed 10 min later to determine the results, as demonstrated in Figure 1B. If no or undetectable concentration was present in the sample, the antigen on the T-line bound to most of the gold standard antibodies and the colors of the T and C lines were all red. With the concentration of DIFE in the extract increasing gradually, the DIFE in the extract reacted with the gold standard antibodies preferentially, making the T-line color gradually lighter and the C-line color gradually darker, and when the C-line color was significantly darker than that of the T-line, the result was judged as positive; If the C-line did not show color, the test strip was invalid.

### 2.3 Sample testing

It was found in the pre-experiment that the CB solution was more suitable as a buffer for detecting pesticide residues in citrus, and it was particularly necessary to design a rational pre-treatment method due to the large difference in odor, rich pigmentation, high moisture content, and the great influence of sample matrix in citrus samples. In this experiment, six pre-treatment methods were designed using extraction solution, two-fold dilution, and centrifugation, from which the pre-treatment method with the highest inhibition rate was selected as the optimal pre-treatment method.

### 2.4 Test strip performance evaluation

#### 2.4.1 Determination of standard curve and detection limit

The blank citrus was used as the sample to add the DIFE standard, which was diluted by extract and configured into 11 gradients of spiked samples of 0, 0.01, 0.02, 0.03, 0.06, 0.15, 0.3, 0.6, 1.2, 1.5, and 1.8 mg/kg, and titrated on the test strips after pre-treatment. The standard curve was developed by reading the test strips after the reaction, and the interval with a good gradient in the standard curve was selected to make the quantitative curve. The final added concentrations of DIFE in citrus blank samples were 0, 0.1, 0.2, 0.3, 0.5, and 1 mg/kg, and each level was repeated five times. The samples were extracted according to the method in Section 1.3.1, and the concentration at which the C-line showed significantly darker than the T-line in the naked eye test was the naked eye detection limit of the test strip.

#### 2.4.2 Specificity and stability evaluation

Triadimenol, Triadimefon, Tebuconazole, Cyclobutanil, and other common triazole pesticides were configured to 10 mg/kg and tested with test strips to observe the color development of C and T lines to determine whether the test strips showed cross-reactivity.

The prepared test strips were stored at 45°C, 37°C, and 4°C and taken out for testing 30 days later. The stability of the test strips was determined by observing the test results.

#### 2.4.3 Comparison experiments

The blank samples were prepared into 20 positive samples containing different concentrations of DIFE, which were mixed into other 20 blank samples for testing, and the samples were examined simultaneously by GC–MS/MS and colloidal gold immunochromatography for DIFE residues to determine the differences of the methods.

Another positive sample containing 0.1, 0.2, and 0.6 mg/kg DIFE was prepared, and 10 parallel tests were conducted for each level, and the fortified recoveries and coefficients of variation were calculated from the actual measured values, thereby judging the accuracy of the test strips.

Pre-treatment method of GC–MS method: The samples were processed according to GB23200.8–2016 and determined by gas chromatography–mass spectrometry analyzer (GC–MS).

The validation results were used to evaluate the sensitivity, specificity, accuracy, and consistency of the colloidal gold method with the reference method by Technical Specification for the Evaluation of Rapid Food Testing Methods.

## 3 Results and analysis

### 3.1 Quality identification of colloidal gold

**Figure 2:**
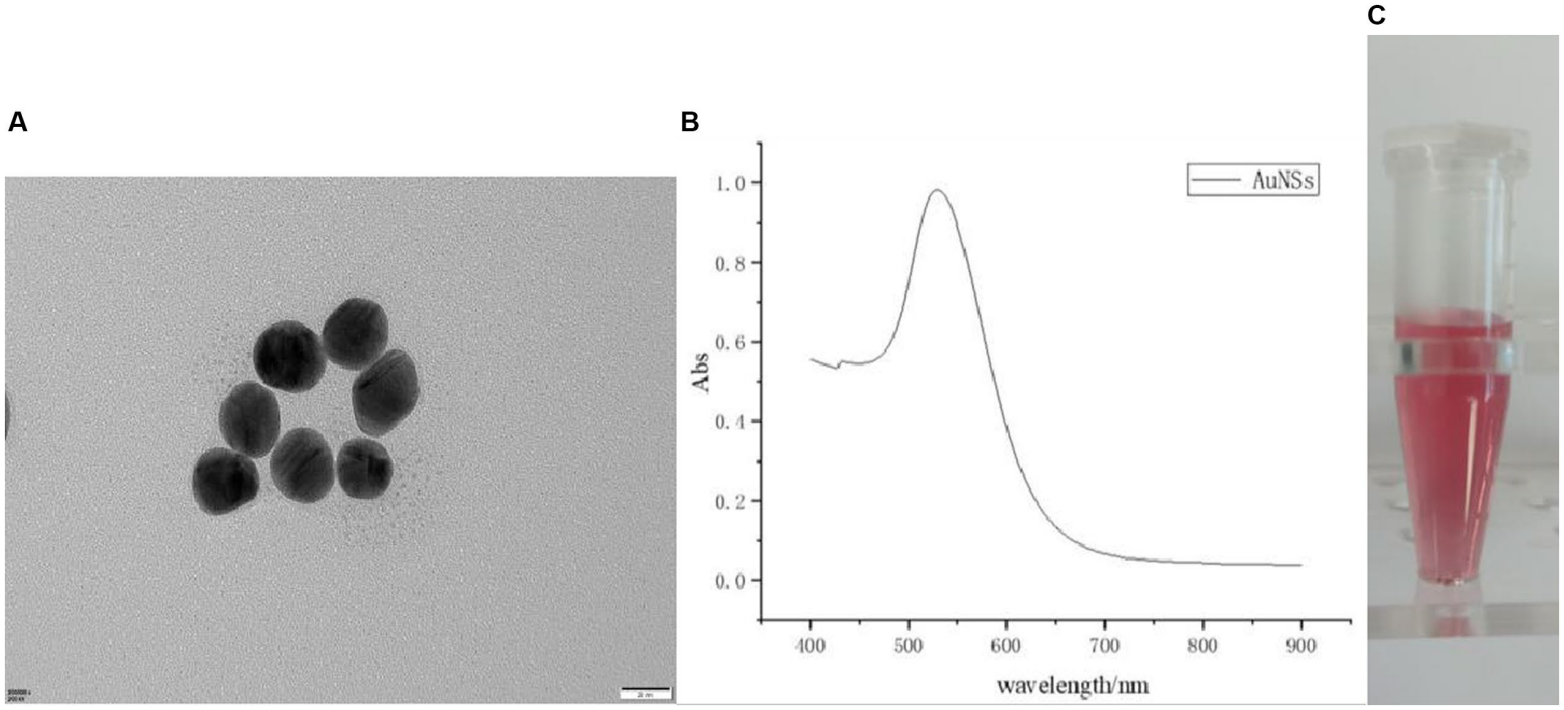
**(A)** Nanogold SEM electron microscopy; **(B)** UV mapping; **(C)** AuNSs solution.

First, by scanning electron microscopy (SEM), it was known that the particles were uniform in size, with a size of 25 ± 2 nm (Figure 2A). After a period of storage at normal temperature, no flocculent substances appeared, indicating that the colloidal gold structure was stable. Second, according to the UV–visible spectrophotometry, the particle size and dispersion state of gold particles determined that the maximum absorption peak was at 524 nm (Figure 2B), and the narrow peak width indicated that the gold particles were well dispersed, and the particle size of the gold particles was about 22 nm by calculation. Finally, the colloidal gold solution was observed by the naked eye, which was pink, clear, and transparent without blocky substance (Figure 2C), illustrating that the colloidal gold was prepared successfully.

### 3.2 Preparation of gold standard antibodies

#### 3.2.1 Determination of the optimal pH for labeling

The initial colloidal gold solution pH was 4.22, with 0.2 mol/L K_2_CO_3_ to adjust the solution pH to 4.22, 5, 6, 7, 8, and 9, respectively. To each tube, 10 μL DIFE antibody was added and mixed. After adding the NaCl solution, the antibody surface charge was massively neutralized, resulting in protein precipitation. In addition, the number of water molecules on the protein surface determined its solution degree. When salt ions were added to the solution, water molecules had a greater affinity for salt ions than proteins, making protein solubility further reduced. If the affinity between gold particles and protein was high and the stable state of the protein was not easily destroyed, there was less protein precipitation phenomenon. After the test, it was found that when pH was 6, the color of the colloidal gold solution tended to be stable without precipitation or discoloration, so the optimal pH in this experiment was 6.

#### 3.2.2 Determination of the optimal labeling amount

Similarly, using the salinization method, 0.5, 1, 1.5, 2, 2.5, and 3 μL of DIFE antibodies were added, respectively. When there were more antibodies in the solution that could not be adsorbed on the surface of gold particles, the phenomenon of protein precipitation aggregation would also occur. It was determined that the solution color tended to be stable when the addition of antibody was 1.5 μL/mL. Therefore, the optimal amount of antibody labeling was 1.5 μL/mL.

#### 3.2.3 Identification of gold standard antibodies

**Figure 3:**
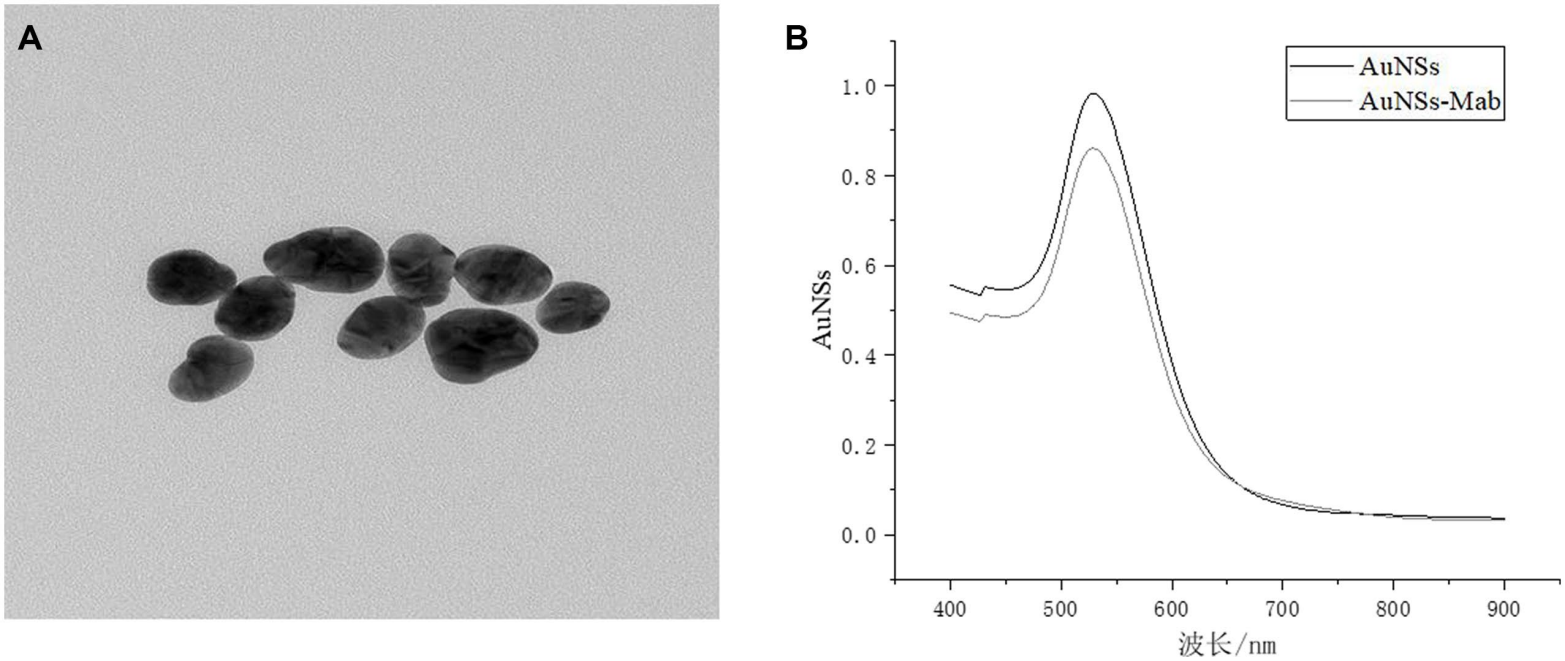
**(A)** Antibody labeling SEM; **(B)** UV spectrum before and after labeling.

As can be seen by SEM (Figure 3A), successful labeling could be judged due to the non-uniform particle size caused by the selective attachment of antibodies to the gold particles in different crystal orientations during the coupling process. It can be seen from Figure 3B that the wavelength of the gold particles labeled with antibody was shifted, and the change of the maximum absorption peak implied the change of the structure of the material to be tested, indicating that the protein and gold particles interacted with each other, and the protein adhered to the surface of the gold particles, resulting in the change of the refractive index, from which the gold standard antibody labeling could be preliminarily proved successful.

#### 3.2.4 Reaction method

It can be seen from the table that the inhibition rate of the lyophilized gold form of the test strip was 91.47% when the concentration of the standard was 0.1 mg/kg, which was higher than that of the sprayed gold form (78.77%), explaining that the binding rate of the gold standard antibody to the antigen in the extract was higher; therefore, lyophilized gold was chosen as the reaction form of the test strip.

### 3.3 Test strip performance test

#### 3.3.1 Determination of sample pre-treatment method

A total of 2 g blank citrus samples were accurately weighed into a 10 mL centrifuge tube, and the extraction solution was added to set the concentration as 0.2 mg/mL of DIFE spiked samples. The samples were prepared according to Table 2, and 100 μL extraction solution was taken for double parallel test strip assay, thus taking the optimal reaction conditions. After calculation, 2 g citrus samples were extracted by adding 2 mL of the CB solution, mixed by vortex for 1 min, put into a centrifuge, 3,000 r/min for 5 min, and 1 mL of the supernatant plus 3 mL extraction solution was taken as the optimal pre-treatment method.

**Table 2:** Pre-treatment methods for citrus samples (*n* = 3).

Sample Pre-treatment Method	Blank citrus samples T/C values	Spiked samples T/C values	Inhibition rate
CB solution (8 mL)	1.845 ± 0.07	1.150 ± 0.02	37.67%
CB solution (8 mL) + centrifuged	3.392 ± 0.11	1.390 ± 0.09	59.02%
CB solution (4 mL) + 2 mL the supernatant plus 2 mL extraction solution	3.097 ± 0.18	1.164 ± 0.04	62.41%
CB solution (4 mL) + centrifuged	1.562 ± 0.07	0.523 ± 0.04	66.52%
CB solution (2 mL) + 1 mL the supernatant plus 3 mL extraction solution	2.757 ± 0.06	0.796 ± 0.02	71.13%
CB solution (2 mL) + centrifuged +1 mL the supernatant plus 3 mL extraction solution	2.925 ± 0.12	0.542 ± 0.05	81.47%

#### 3.3.2 Quantitative curve and detection limit analysis

**Figure 4:**
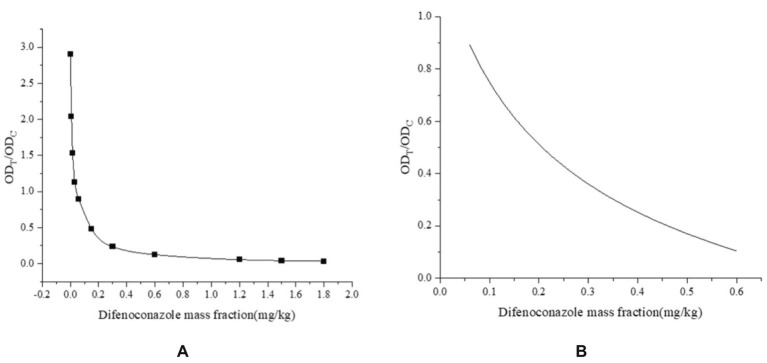
**(A)** 0–1.8 mg/kg DIFE standard curve; **(B)** 0.06–0.6 mg/kg DIFE fitting curve.

The LOD is the minimum concentration or amount of a substance to be measured that can be detected from a sample by a particular analytical method within a given confidence level. The standard curve was drawn by double parallel test strip assay with 10 different gradients of mass fractions of DIFE as the horizontal coordinates and the T/C values of the test strip instrument as the vertical coordinates, as shown in Figure 4A. The interval with a good linear range in the standard curve was selected for curve fitting, as shown in Figure 4B, 0.06–0.6 mg/kg was used for linear fitting, and the regression equation was *y* = −0.3449x + 2.2796, *R*^2^ = 0.9906, and a quantitative detection range of 0.06–0.6 mg/kg.

**Figure 5:**
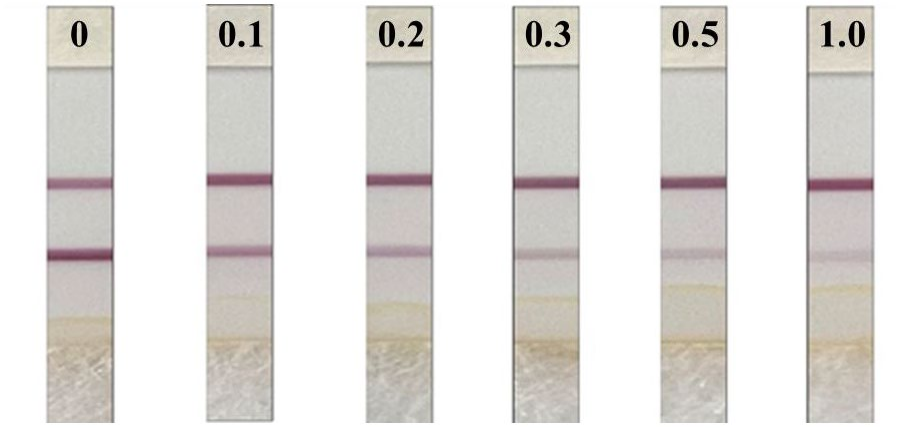
Detection limit of DIFE test strips.

The test results are shown in Figure 5. The citrus substrate had no significant effect on the test strip after 3-fold dilution, and the minimum concentration of the C-line significantly deeper than the T-line was 0.2 mg/kg, so the naked eye detection limit of the test strip was determined to be 0.2 mg/kg, that is, when the sample concentration in citrus was ≥0.2 mg/kg, the test strip was positive and the general test strip OD_T_/OD_C_ was ≤0.6.

#### 3.3.3 Specificity and stability analysis

**Figure 6:**
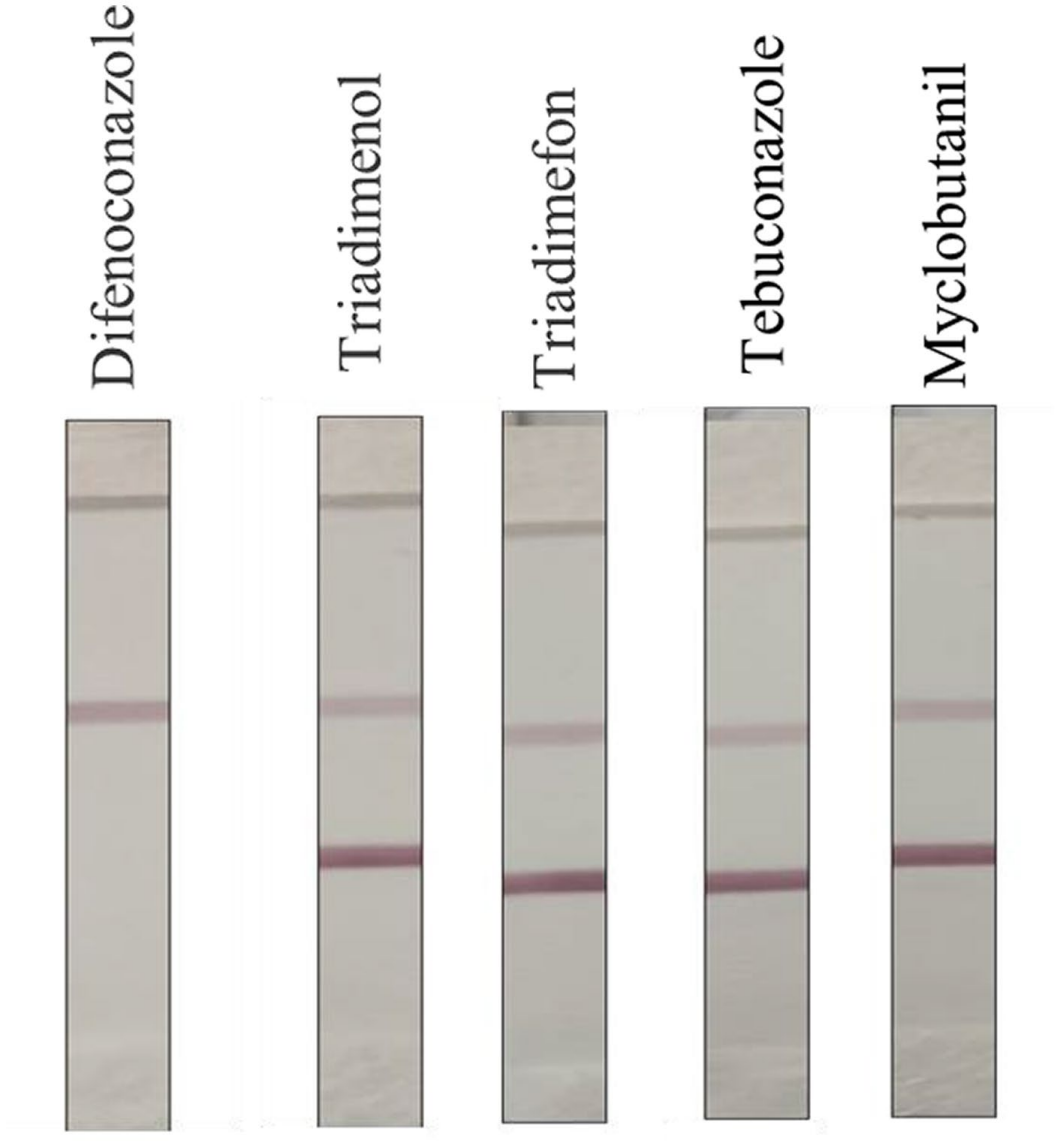
Specificity of DIFE test strips.

As shown in Figure 6, after the cross-reaction test, it was found that there was no significant cross-reaction between DIFE and other commonly used triazole pesticides, and the test strip results were all negative. Because the concentration of the DIFE standard was too high, the gold-labeled antibody was sufficient with the antigen in the standard, and the gold-labeled antibody lacked the binding site with the T-line antigen, and the T-line was not colored. Therefore, the test strips could be considered to have good specificity.

**Figure 7:**
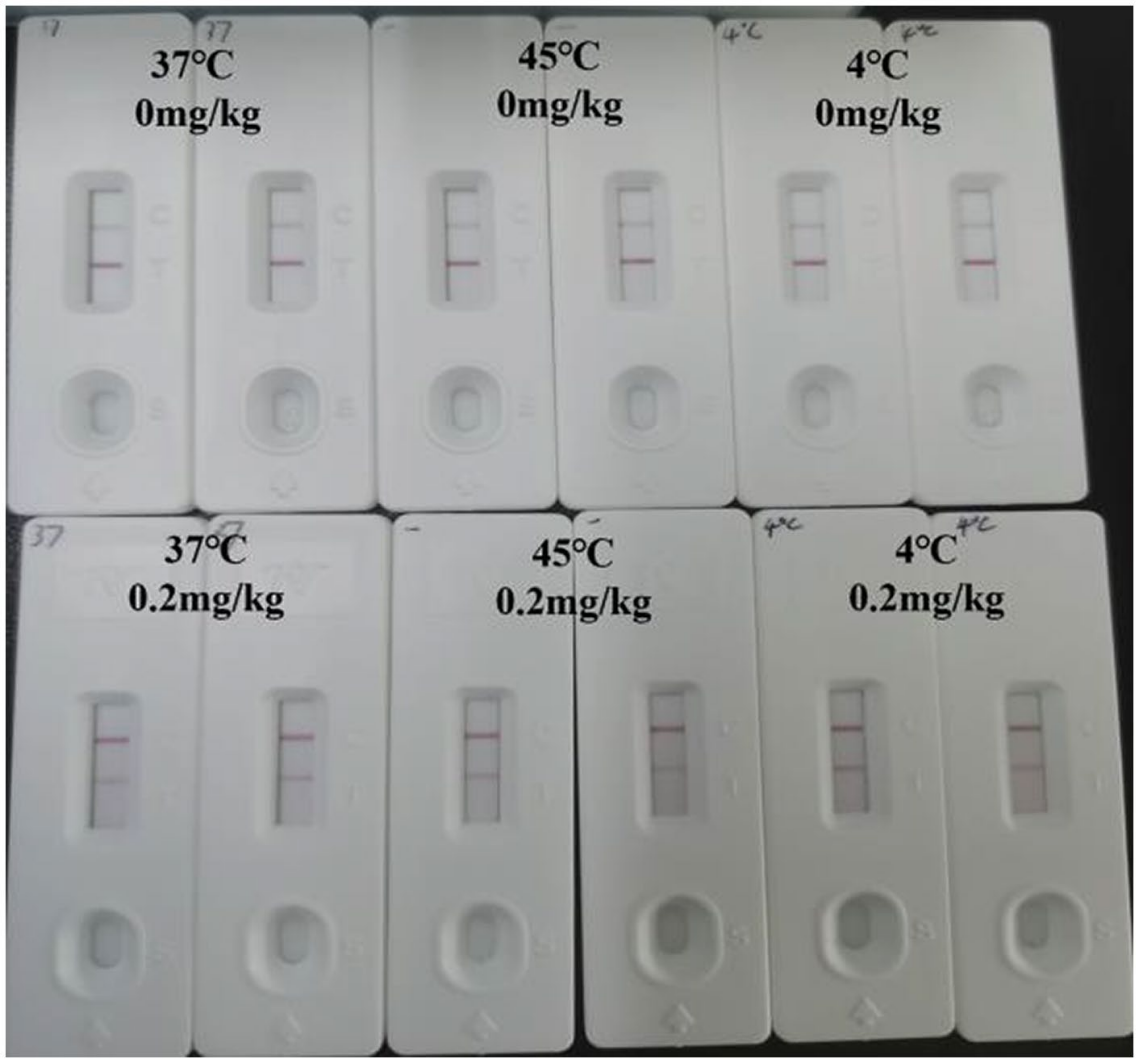
Stability test.

After 30 days of destructive experiments, the final measured results are shown in Figure 7. After being stored at three temperature conditions for 30 consecutive days, the C-line showed color indicating that the test strips were still effective and the results were consistent, explaining that the test strips were stable and could be used for long-term storage at room temperature.

#### 3.3.4 Comparison experiments

The results of 10 parallel tests for each spiked level of the same batch of test strips are shown in Table 3. The spiked recoveries of the prepared test strips were in the range of 66.5–76%, and the coefficients of variation were 8.92–11.94%, which met the assay requirements.

**Table 3:** Spiked recoveries and coefficients of variation of test strips (*n* = 10).

Addition concentration (mg/kg)	GICA			GC–MS		
	T/C values (average)	Coefficient of variation (%)	Spiked recovery rate (%)	Average (mg/kg)	Coefficient of variation (%)	Spiked recovery rate (%)
0.1	0.073 ± 0.01	10.86	73	0.080 ± 0.01	9.35	80
0.2	0.133 ± 0.01	8.94	66.5	0.168 ± 0.02	6.65	84
0.6	0.456 ± 0.05	11.94	76	0.594 ± 0.05	7.83	99

**Figure 8:**
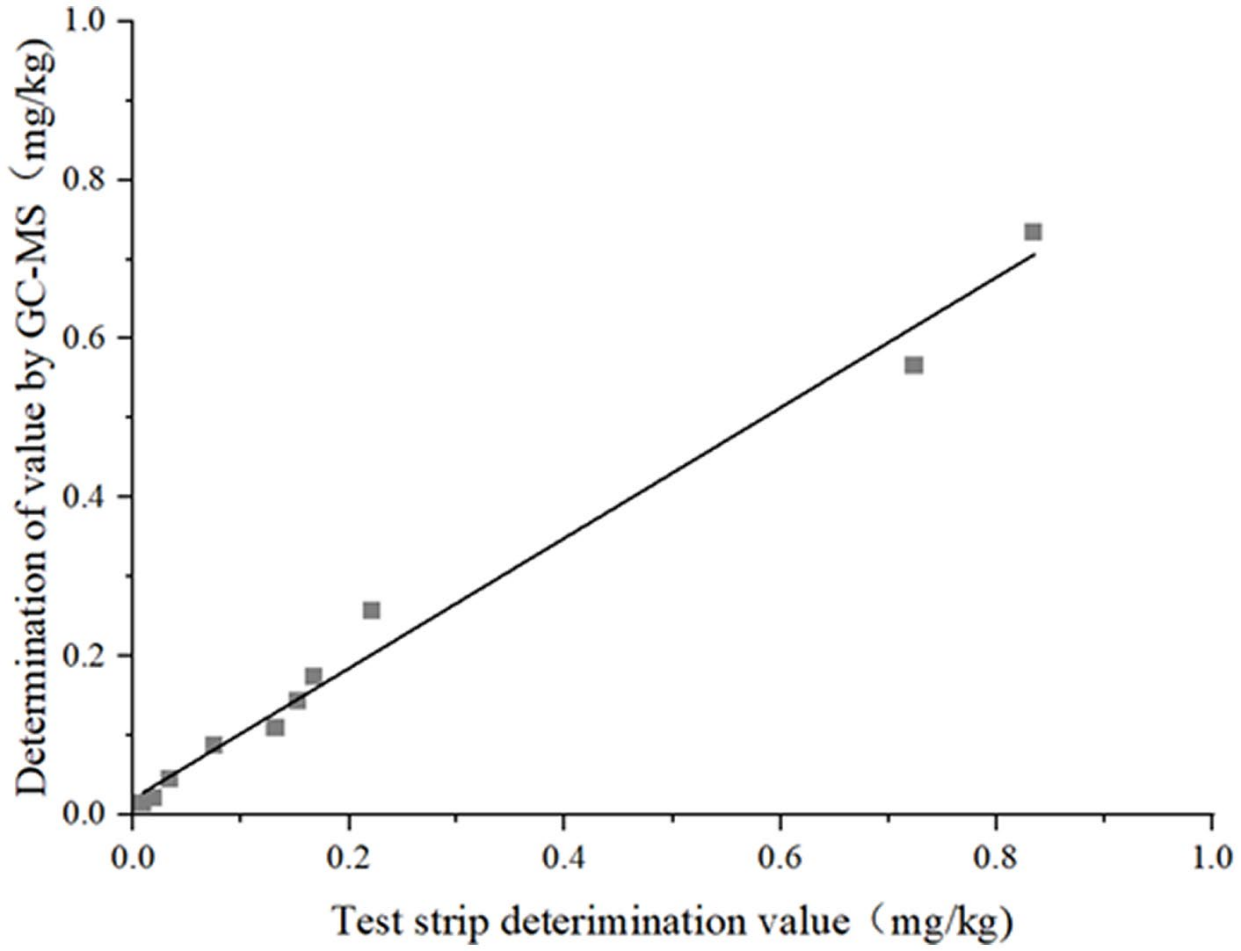
Correlation of test results of orange by test strip and GC–MS.

Comparison experiment results: Among the 40 prepared samples, 10 were randomly selected for blind testing, whose results are shown in Table 4. Within the limit of quantitation, the relative deviation of the two results was in the range of 4.02–27.69%, with a false positive rate of 0 and a false negative rate of 0. The correlation analysis of the two test results is shown in Figure 8. The linear equation of the test strip and the GC–MS test results are *Y* = 1.2022x − 0.022, *R*^2^ = 0.9864, with good linear correlation. The test results showed that the results of the well-prepared colloidal gold immunochromatographic test strips for DIFE were accurate and reliable, and could be well applied to the screening of DIFE residues in citrus.

**Table 4:** Comparison of blind test results of colloidal gold and GC–MS samples (*n* = 3).

Sample number	GC–MS measured values (mg/kg)	Test strip measurement value (mg/kg)	Relative Deviation (%)
1	0.014 ± 0.008	0.009 ± 0.006	35.71
2	0.023 ± 0.008	0.019 ± 0.007	17.39
3	0.143 ± 0.02	0.135 ± 0.01	5.59
4	0.174 ± 0.02	0.167 ± 0.01	4.02
5	0.567 ± 0.09	0.724 ± 0.05	27.69
6	0.734 ± 0.07	0.835 ± 0.06	13.76
7	0.109 ± 0.004	0.132 ± 0.01	21.10
8	0.045 ± 0.007	0.034 ± 0.006	24.44
9	0.087 ± 0.006	0.075 ± 0.008	13.79
10	0.258 ± 0.02	0.221 ± 0.05	14.34

## 4 Conclusion

In this experiment, a quantitative and rapid method for determining DIFE residues in citrus has been established. Compared with the detection limit of 0.5 mg/kg of the test strip created by Tingting Cui for the detection of DIFE, through the freeze-dried gold method, this method increased the detection limit by 2.5 times, resulting in a detection limit of the test strip at 0.2 mg/kg, a quantitative detection range of 0.06–0.6 mg/kg, and a reaction time of 10 min, which can meet the national standard for the detection of DIFE residues in citrus fruits. Compared with the GC–MS, the test strip was accurate, reliable, and had good specificity. No organic reagent was added to the extract, which prevented environmental pollution and was friendly to the environment. In the future, the sample extraction solution will be further explored, the extraction efficiency of DIFE and the pre-treatment method will be optimized, and the test strips with more convenient operation and higher sensitivity will be prepared.

## Data availability statement

The original contributions presented in the study are included in the article/supplementary material, further inquiries can be directed to the corresponding authors.

## Author contributions

RW: Writing – original draft, Writing – review & editing. MG: Writing – review & editing, Writing – original draft. YaL: Data curation, Writing – original draft. WZ: Software, Writing – original draft. KZ: Methodology, Writing – original draft. YZ: Validation, Writing – original draft. CY: Investigation, Writing – original draft. YuL: Funding acquisition, Resources, Writing – review & editing. JW: Funding acquisition, Resources, Writing – review & editing. YW: Funding acquisition, Methodology, Resources, Visualization, Writing – review & editing.
